# Engineered exosomes: a potential therapeutic strategy for septic cardiomyopathy

**DOI:** 10.3389/fcvm.2024.1399738

**Published:** 2024-06-28

**Authors:** Lixia Mao, Songtao Liu, Yongxia Chen, Huiyi Huang, Fenghua Ding, Liehua Deng

**Affiliations:** ^1^ Department of Critical Care Medicine, Affiliated Hospital of Guangdong Medical University, Zhanjiang, China; ^2^ Outpatient Appointment Center, Affiliated Hospital of Guangdong Medical University, Zhanjiang, China

**Keywords:** sepsis, septic cardiomyopathy, engineered exosomes, extracellular vesicles, drug delivery

## Abstract

Septic cardiomyopathy, a life-threatening complication of sepsis, can cause acute heart failure and carry a high mortality risk. Current treatments have limitations. Fortunately, engineered exosomes, created through bioengineering technology, may represent a potential new treatment method. These exosomes can both diagnose and treat septic cardiomyopathy, playing a crucial role in its development and progression. This article examines the strategies for using engineered exosomes to protect cardiac function and treat septic cardiomyopathy. It covers three innovative aspects: exosome surface modification technology, the use of exosomes as a multifunctional drug delivery platform, and plant exosome-like nanoparticle carriers. The article highlights the ability of exosomes to deliver small molecules, proteins, and drugs, summarizing several RNA molecules, proteins, and drugs beneficial for treating septic cardiomyopathy. Although engineered exosomes are a promising biotherapeutic carrier, they face challenges in clinical application, such as understanding the interaction mechanism with host cells, distribution within the body, metabolism, and long-term safety. Further research is essential, but engineered exosomes hold promise as an effective treatment for septic cardiomyopathy.

## 1 Introduction

Sepsis is a life-threatening organ dysfunction caused by a dysregulated host response to infection (1). Septic cardiomyopathy is a prevalent complication of sepsis unrelated to ischemia, and has a high fatality rate (2). It commonly presents with symptoms such as ventricular dilation, reduced contractility and/or ventricular dysfunction, coupled with a diminished responsiveness to volume infusion (3, 4). Current treatment strategies primarily involve supportive care, including antibiotics and hemodynamic improvement (5, 6). Potential risk factors for septic cardiomyopathy encompass pathogen-associated molecular patterns (PAMPs), cytokines, and nitric oxide (7). The exact pathogenesis remains unclear, highlighting the need for more effective and safer therapeutic approaches for septic cardiomyopathy.

Extracellular vesicles (EVs), which are nanoscale lipid bilayer membrane structures secreted by cells (8), can be divided into two main categories based on their generation mechanisms: endosome-origin exosomes and plasma membrane-derived ectosomes (9, 10). Exosomes, as naturally occurring extracellular vesicles with diameters ranging from 30 to 200 nm, have garnered considerable interest in recent years. Found in various biological fluids, almost all eukaryotes or prokaryotes can release, these vesicles that carry a range of cellular components, including lipids, proteins, DNA, and RNA (11–14). They facilitate intercellular communication by being taken up by distant cells, influencing the function and behavior of recipient cells (15). Given their excellent biocompatibility, inherent stability, low-immunogenicity, and capacity for targeted delivery and immunomodulation (16, 17), exosomes have been as therapeutic agents for various diseases' studies and treatment (18–20).

However, naturally occurring exosomes carry different cargos, and not all of them are effective in treating diseases. For instance, certain cargoes like miR-1249-3p are known to alleviate insulin resistance and inflammation in a type 2 diabetes mouse model (21). Similarly, miR-144-3p can inhibit the growth, migration, and invasion of osteosarcoma cells (22), while miR-150-5p serves as a negative regulator of disease severity (23), thereby slowing disease progression. Conversely, some cargoes, such as miR-155-5p, contribute to severe acute pancreatitis-related intestinal barrier damage (24). miR-30d-5p induces macrophage M1 polarization and triggers macrophage pyroptosis, playing a significant role in sepsis-related acute lung injury (25). Additionally, miR-423-5p promotes cancer growth and metastasis and can be a potential diagnostic and prognostic marker for gastric cancer, posing potential risks to the body (26). Therefore, it is necessary to carry out certain modifications to exosomes to weaken their side effects and enhance the therapeutic function. At the same time, we can also modify the surface of exosomes so that they can target the heart and stay in the damaged heart for a longer period, making their therapeutic effect more significant. Research on engineered exosomes has shown their critical role in modulating the immune response to inflammation (27, 28). Furthermore, they contribute to cardiac protection by preventing cardiomyocyte apoptosis (29, 30), boosting mitochondrial function, and preserving myocardial contractility (31). Engineered exosomes hold considerable promise as a novel treatment strategy for septic cardiomyopathy, highlighting the need for comprehensive investigative efforts in this area.

This article systematically reviews the pathophysiology of septic cardiomyopathy, the characteristics of engineered exosomes, and the connection between them. The article also discusses different construction strategies of engineered exosomes in protecting cardiac function and treating septic cardiomyopathy, and analyzes the challenges faced in the development and utilization of engineered exosomes, aiming to provide reference and inspiration for future research.

## 2 Materials and methods

We systematically searched the PUBMED database and manually scanned the reference lists of articles. We conducted a search of the PubMed database for the most relevant articles regarding engineering exosomes, sepsis, septic cardiomyopathy, and the relationship between septic cardiomyopathy and engineering exosomes. For the search formula, we used the following terms: “(((exosomes)OR (extracellular vesicle) OR (EVs))) AND ((sepsis)OR (septic))”, “engineering exosomes”, “sepsis and EVs”, “miRNA and exosomes”, “septic cardiomyopathy”, “plant exosomes”. Initially, the PubMed database showed 594 results. In this study, a thorough evaluation of article titles was conducted to ascertain the inclusion of at least one relevant search term. Articles that did not satisfy the inclusion criteria or focused on subjects divergent from the treatment of engineering exosomes in septic cardiomyopathy were methodically excluded. Consequently, the final analysis incorporated 148 studies. The schematic diagram of the article was produced using Microsoft Office PowerPoint software. The research was conducted from a holistic viewpoint, concentrating on the treatment of engineering exosomes in sepsis.

## 3 Pathophysiology of septic cardiomyopathy

Septic cardiomyopathy develops from a disordered immune response to infection, a process that involves pathogen-associated molecular patterns (PAMPs) and injury-associated molecular patterns (DAMPs) that activate pattern recognition receptors and triggers a variety of intracellular pathways, such as NF-κB and mitogen-activated protein kinase pathways. Septic cardiomyopathy presents as an inflammatory state, with evidence of inflammatory cell infiltration in affected organs (32).

The field of septic cardiomyopathy research has some puzzling phenomena. Earlier studies indicated increased cardiac output in sepsis patients, suggesting that cardiac systolic function remained intact. Yet, further research revealed a reduction in left ventricular ejection fraction (EF) in these individuals (33). Intriguingly, having a reversible decrease in EF correlates with improved prognoses in contrast to maintaining stable EF levels (34). Numerous studies are exploring the mechanisms behind myocardial dysfunction in sepsis. These studies cover various aspects, such as the emergence of circulating myocardial inhibitory substances, the weakening of adrenergic pathways, the production of nitric oxide and reactive oxygen species, abnormal calcium regulation, mitochondrial dysfunction, disturbances in the coronary microvasculature, and the suppression of genetic expression for sarcomeric and mitochondrial proteins.

Patients with sepsis exhibit a non-ischemic myocardium, supported by elevated plasma troponin levels (35, 36). Studies have identified “myocardial inhibitory substances” in circulation, such as tumor necrosis factor and IL-1β, which can suppress cardiomyocyte function (37, 38). The formation of S-nitroso albumin from DAMPs and nitric oxide (NO), along with various other circulating mediators, may also contribute to the pathogenesis of septic cardiomyopathy. Sepsis-induced PAMP and DAMP signaling can initiate an inflammatory cascade via Toll-like receptor activation (39). This activation may enhance cytokine production, which can directly inhibit cardiomyocyte contraction (40). These cytokines activate inducible nitric oxide synthase (iNOS), resulting in an overproduction of NO that causes vasodilation and hypotension (41). Oxidative stress, implicated in septic cardiomyopathy, can damage myocardial cell membrane lipids, proteins, and DNA, leading to cellular dysfunction and death. Free radical scavengers have been found to improve cardiac function in sepsis mouse models (42). Overstimulation of sympathetic nerves negatively impacts myocardial performance and contractility. Inflammation-related calcium responsiveness impairment may result in myocardial contractile dysfunction (43). Severe mitochondrial dysfunction in sepsis is strongly associated with poor outcomes (44). During sepsis, local disturbances of the cardiac microcirculation may trigger a compensatory metabolic closure in the region of hypoperfusion, leading to abnormal cardiac function and energy (45). Although septic cardiomyopathy may be very severe, it is usually reversible for surviving patients (46).

Treatment of septic cardiomyopathy faces limitations, primarily focusing on managing sepsis itself through infection control, fluid resuscitation, and vasoactive drugs to maintain hemodynamic stability. Therefore, we still need the development of more comprehensive, safe, and effective treatment strategies for septic cardiomyopathy.

## 4 Overview of exosomes

### 4.1 Exosome biogenesis

Cellular secretion includes a spectrum of extracellular vesicles, with exosomes and ectosomes, each characterized by their unique origins. Exosomes are extracellular vesicles derived from endosomes with a diameter between 30 and 200 nm. The genesis of endosomes is traced back to the plasma membrane's internal budding. Exosomes are composed of proteins from the plasma membrane and Golgi apparatus, along with lipid and nucleic acid contents, encompassing cytokines, molecular patterns linked to pathogens and damage, and autoantigens. The process of segregating proteins and RNA into exosomes is stringently controlled, enabling cells to emit exosomes with varied properties based on the molecular cues triggering their synthesis. Late-stage endosomes within multivesicular bodies fuse with the cell's outer membrane, releasing their cargo, termed exosomes. Lysosomes can lead to the breakdown of multivesicular bodies. Subsequently, recipient cells internalize exosomes, which then move the activated receptors to the cell's surface or attach to these receptors to initiate signaling pathways (47). Exosome-transported mRNA can be converted into protein, while exosome-delivered miRNA can specifically target mRNA expression in the recipient cells (48).

### 4.2 Overview of the engineered exosomes

**Figure 1:**
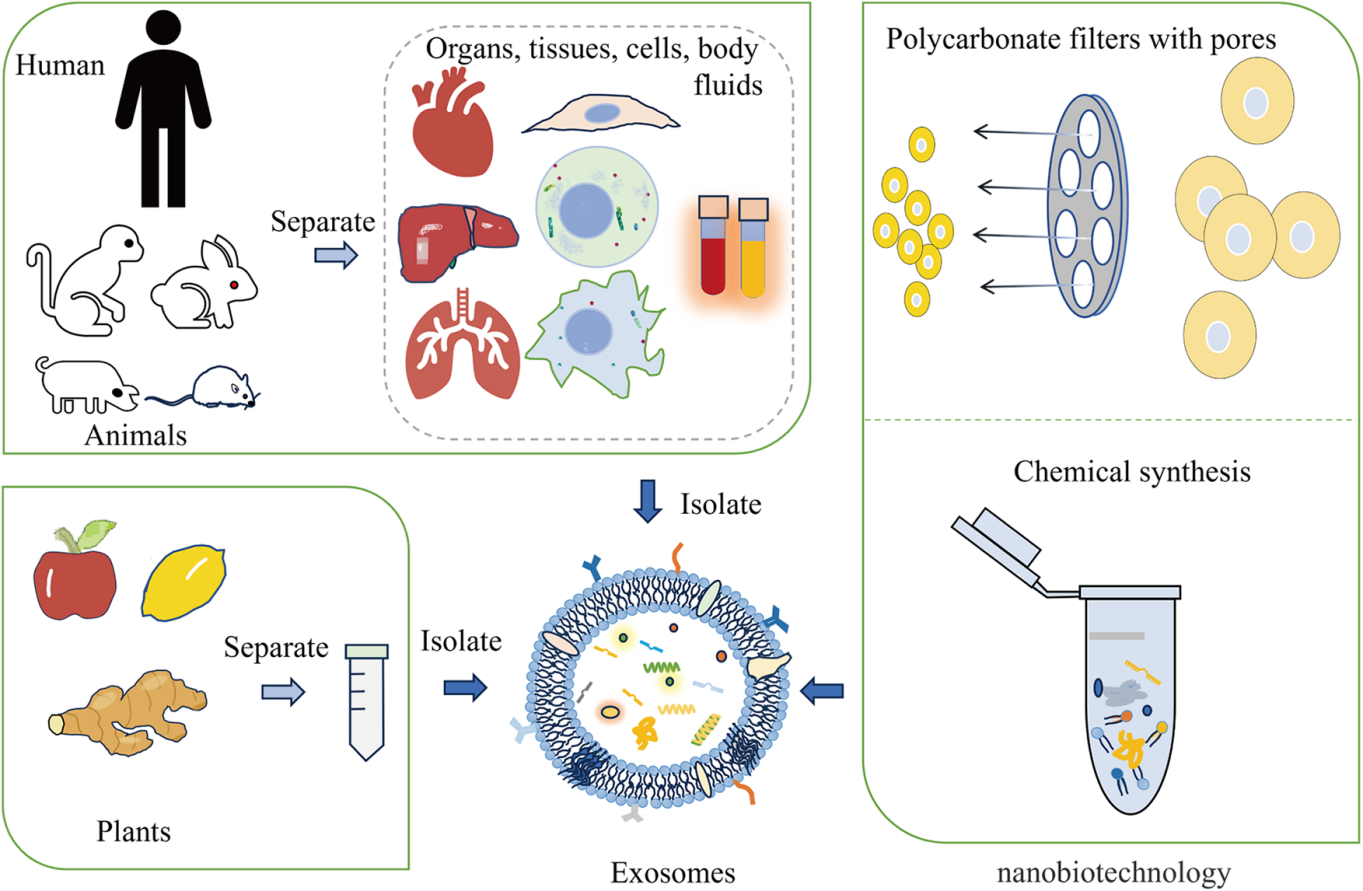
The main origins of exosomes include animals, plants, and nanobiotechnology techniques. Exosomes can be derived from animal organs, tissues, cells, and body fluids. Additionally, plants like apples, lemons, and ginger are capable of providing exosomes. Nanobiotechnology techniques, such as forcing cells through membrane pores or using supramolecular chemistry methods, can also generate synthetic exosomes.

Engineered exosomes preserve the inherent properties of natural exosomes while acquiring additional or enhanced functions through bioengineering. These enhancements may involve targeting particular diseased tissues or cells or encapsulating specific drugs or functional RNA (49). Exosomes are primarily derived from animals, plants, and artificial synthesis. The modification of exosomes can occur via genetic engineering, chemical modifications (both covalent and non-covalent), alterations to the cell membrane, and encapsulation with biomaterials (50, 51) (Figure 1). For instance, autologous exosomes can be engineered to carry specific ligands, enabling the stable delivery of therapeutic agents (52). Exosome-loaded drugs mainly include cell transfection, direct co-incubation, sonication, electroporation, freezing and thawing, and extrusion. When compared to other biological treatments like cell and viral therapies, exosomes have the advantage of not being able to divide or replicate. This characteristic could make engineered exosome therapies comparatively safer in terms of tumorigenicity and infectivity (53–55). Furthermore, engineered exosomes have been demonstrated and well tolerated without significant side effects. Furthermore, engineered exosomes are well tolerated in without significant side effects. For instance, the study conducted by Bellavia et al. demonstrated the ability of exosomes released by HEK293T cells (Exo or IL3l-Exo) loaded with or without Imatinib to reduce tumor growth tested in an *in vivo* tumor xenograft model (56). Another toxicological study of MSC-Exo showed that MSC-Exo were safe without significant side effects when topical treatment on skin (57). In addition, the result of a phase I study showed that autologous dendritic cell (DC)-derived exosomes (DEX) loaded with MAGE tumor antigen were well tolerated in patients with non-small cell lung cancer (NSCLC) without evidence of severe toxicity (58).

## 5 The link between exosomes and septic cardiomyopathy

Exosomes may be linked to septic cardiomyopathy. Initially considered mere cellular waste disposal entities, they are now recognized as nanoscale intercellular communication carriers. Janiszewski et al. observed a 60% increase in platelet-derived exosomes in the plasma of septic patients compared to healthy controls. These exosomes exhibit pro-apoptotic NAD(P)H oxidase activity and can produce reactive oxygen species (ROS) via NADPH oxidase. An abundance of exosomes can lead to endothelial damage and potentially affect nearby cardiomyocytes, resulting in cardiac dysfunction (59).

Azevedo et al. analyzed blood samples from 55 septic shock patients and 12 healthy individuals. The research revealed that exosomes derived from platelets in patients with sepsis markedly reduced the myocardium contractility and hindered its function in isolated rabbit hearts (60). The NLRP3 inflammasome, activated by TXNIP, has been studied by Wang et al. They demonstrated that TXNIP-NLRP3 complexes embed in CD63 exosomes and transfer from monocytes to resident cardiac macrophages. These complexes trigger the activation of caspase-1, leading to the cleavage of IL-1β and IL-18 precursors. This process results in the secretion of the active forms of IL-1β and IL-18, contributing to the dysfunction of the inflammatory response observed in sepsis (61). Moreover, exosomes play additional roles. Zhou et al. proposed that exosomes of human mesenchymal stem cells (MSC) carry large amounts of mRNA that provides the protein PINK 1 needed to avoid calcium overload. Upon intraperitoneal injection of human MSC-derived exosomes, these mRNAs can be transported to cardiomyocytes, enhancing PINK1 expression, restoring calcium regulation, and improving myocardial function (62).

Exosomes demonstrate considerable potential in disease diagnosis (63–66). Released by nearly every cell type, these entities transport numerous molecules and are found in all types of bodily fluids. Exosomes are detectable in liquid biopsy samples, such as blood, urine, and cerebrospinal fluid. Undoubtfully, exosomes for diagnostic purposes have attracted widespread attention. Some of the identified miRNAs, for instance, miR-150-5p, miR-125b, and miR-495, may be potential markers of cardiomyopathy (23, 67, 68), and their aberrant expression is closely associated with cardiac inflammation and injury (69). Wang's study involving 214 sepsis patients assessed serum miRNAs, identifying six with significant expression differences between survivors and non-survivors: miR-223, miR-15a, miR-16, miR-122, miR-193b*, and miR-483-5p. It was observed that miR-15a, miR-122, miR-193b*, and miR-483-5p were expressed at significantly higher levels in non-survivors, while the expression of miR-223 and miR-16 decreased. Notably, miR-193b* showed a higher predictive value for sepsis mortality than SOFA scores and APACHE II scores (70). Currently, liquid biopsy methods for detecting extracellular vesicle contents are utilized in prostate cancer diagnosis (71). Similarly, studies on sepsis have demonstrated that mRNA, miRNA, or proteins found in exosomes can be potential biomarkers (Supplementary Table S1). Consequently, the detection of these miRNA can augment the specificity of early septic cardiomyopathy diagnosis.

## 6 Exosomes for the treatment of septic cardiomyopathy

Engineered exosomes offer significant potential for treating septic cardiomyopathy (Table 1). Firstly, they can home in on the heart, delivering drugs directly to affected areas, and enhancing therapeutic efficacy while minimizing systemic side effects (82–85). Secondly, engineered exosomes can modulate immune responses, potentially inhibiting the cardiac inflammatory response and reducing myocardial damage (86). Moreover, they possess cell-protective and repair capabilities, which could improve cardiac function by promoting cardiomyocyte survival and regeneration (87–89).

**Table 1:** Examples of engineered exosomes constructed in different ways to protect cardiac function.

Exosome origin	Model	Content	Effect
M2 macrophages	Mice with sepsis induced by intraperitoneal injection of lipopolysaccharide (LPS)	miR-24-3p	Improve cardiac function, reduce myocardial cell apoptosis and serum inflammation in myocardial tissue (30).
Mesenchymal stem cells	Mouse with sepsis induced by cecal ligation and puncture (CLP)	miR-223-KO	Drastically intensified the harm induced by sepsis. On the flip side, protective qualities were evident in exosomes sourced from WT-MSCs (72).
Mesenchymal stem cells	Mouse with sepsis induced by CLP	miR-141	Improved heart muscle damage in mice with sepsis by delivering miRNA-141, which modulates the PTEN/β-catenin signaling pathway (73).
Mesenchymal stem cells	LPS-induced cardiomyocytes (H9C2 cells) *in vitro* and mice with sepsis-induced *in vivo*	miR-146a-5p	Offered protection to cardiomyocytes in inflammation models *in vitro* and maintained the integrity of myocardial tissue in sepsis models *in vivo* (74).
Bone marrow stromal cells (BMSCs)	Mouse with sepsis induced by CLP	miR-126	Decreased the sepsis-induced upregulation of adhesion molecules and the influx of immune cells such as macrophages and neutrophils in the cardiac muscle of HSPA12B^–/–^ mice (75).
Human umbilical vein endothelial cells	Mouse with sepsis induced by CLP	HSPA12B	Downregulated the NF-κB pathway, thereby reducing mortality and complications of septic cardiomyopathy (76).
HEK293T cells	LPS endotoxemia mouse model and CLP sepsis mouse model	super-repressor IκB (srIκB)	Combated inflammatory responses, thereby ameliorating pro-inflammatory cytokine storm and subsequent organ damage (77).
Cardiac progenitor cells	H2O2-induced H9C2 cell	miR-21	Reduced cardiomyocyte apoptosis (29).
Mesenchymal stem cells	Mouse with myocardial infarction	miR-22	Improved the viability of cardiomyocytes and reduced cardiac fibrosis (78).
Mesenchymal stem cells	Mice with MI induced by ischemia-reperfusion	miR-21, CD47	Effectively delivered to cardiomyocytes, resulting in notable anti-apoptotic effects, and reducing cardiac inflammation (79).
HEK293T cells	Mouse with myocardial infarction	miR-21	Prevented cell death via apoptosis, culminating in a notable boost to cardiac functionality (80).
Human cardiosphere-derived cells	Rats and pigs with MI induced by ischemia-reperfusion	miR-181b	Diminishing levels of PKCδ mRNA and providing cardio-protection (81).

### 6.1 Exosome surface modification methods

**Figure 2:**
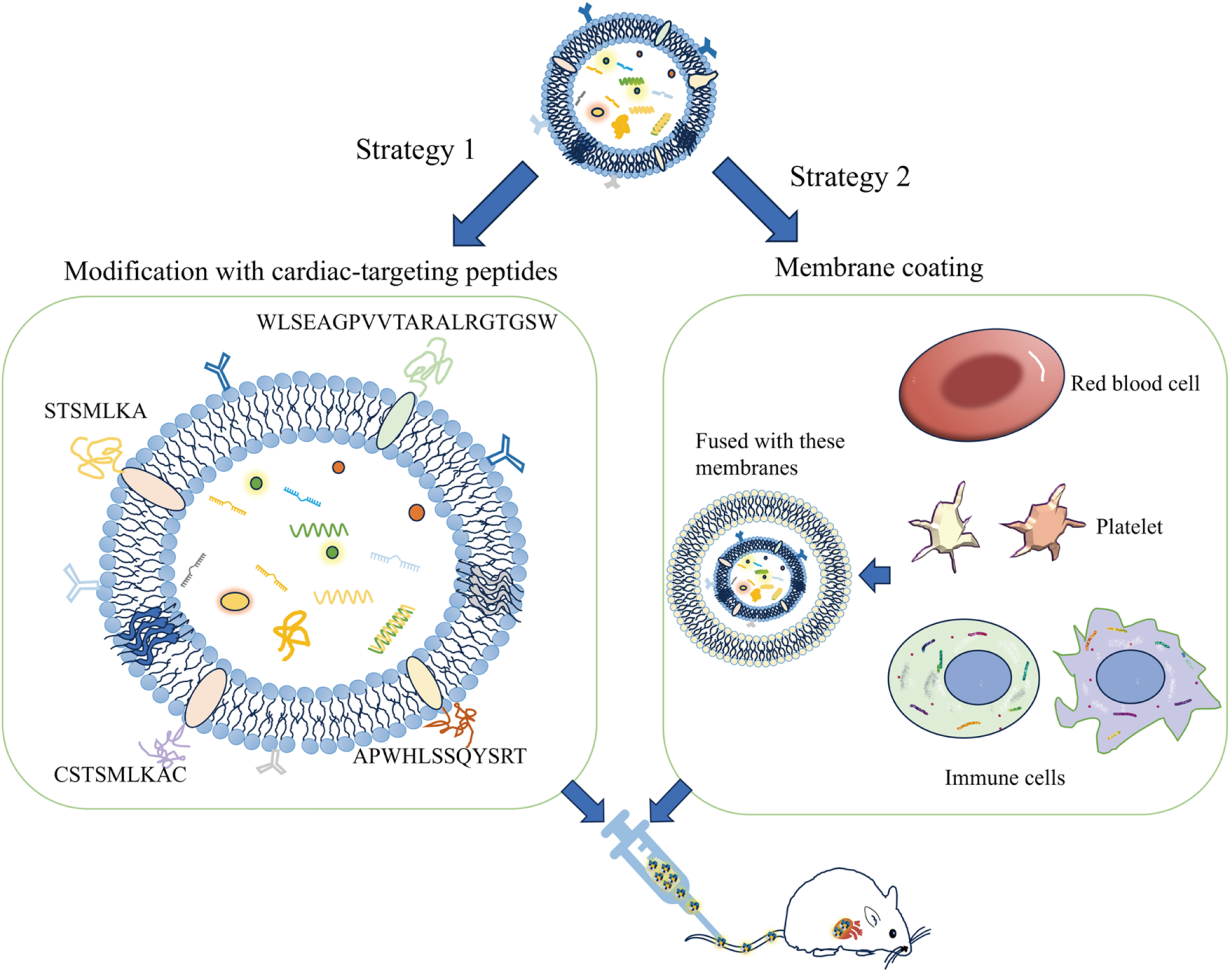
A schematic diagram of the construction of the exosome cardiac targeting system modified with targeting peptides and cell membranes. The cardiac targeting peptides include sequences like WLSEAGPVVTARALRGTGSW, APWHLSSQYSRT, STSMLKA, and CSKTSMLKAC. When these sequences are attached to the exosome membrane, the exosomes can be delivered to cardiomyocytes through intravenous injection. Additionally, exosomes that have fused with the membranes of red blood cells, platelets, or immune cells show a greater tendency to adhere to damaged cardiomyocytes.

Exosomes can be genetically engineered to display specific surface markers by modifying proteins or peptides (Figure 2). For instance, exosomes modified with myocardial-targeting peptides or antibodies can enhance uptake by cardiomyocytes *in vitro*, reduce apoptosis, and increase aggregation in myocardial tissue, thereby improving treatment outcomes (83, 90). Myocardial-targeting peptides such as WLSEAGPVVTARALRGTGSW (91, 92), APWHLSSQYSRT (93, 94), STSMLKA (95, 96), CSKTSMLKAC (84, 85, 97, 98), have specific targeted effects for cardiovascular conditions. Yang et al. discovered that STSMLKA was preserved in the ischemic myocardium after intravenous delivery. This result suggests that the engineered exosome can target the infarcted hearts after non-invasive intravenous injection, which may help with recovery after myocardial infarction (96). In addition, CDCs-EVs were engineered using a DOPE-NHS linker paired with CSTSMLKAC. in one study. By targeting exosomes to the infarcted heart, it could improve fibrosis and increase cell proliferation and angiogenesis (84). Although the exact mechanism of the interaction between CHP and myocardium is unclear, it can amplify the role of exosomes by combining CHP with exosomes. The comparison of the administration methods of engineered exosomes are listed in Supplementary Table S2.

In research by Gupta et al., expressing tumor necrosis factor receptor 1 (TNFR1) and interleukin 6 signaling protein (IL-6ST) on extracellular vesicles significantly mitigated systemic inflammation induced by lipopolysaccharide (LPS). These vesicles with cytokine decoys outperformed clinically approved drugs targeting TNF-α and IL-6 pathways in terms of therapeutic effects (99). Experimental evidence confirms that the CD47-SIRP*α* combination can activate the “don't eat me” signal, thereby inhibiting monocyte phagocytosis. Additionally, exosomes with high CD47 expression can promote immune evasion and extend their circulation half-life in mice (100, 101). MiR21-loaded CD47-EVs were efficiently delivered to cardiomyocytes, resulting in notable anti-apoptotic effects, and reduced cardiac inflammation (79). Since proteins on exosome surfaces are pivotal for biodistribution and cell targeting (102), their modification remains a promising area for ongoing investigation.

Biomimetic nanocarriers coated with cell membranes, or exosomes, leverage the native properties of the cell membrane to achieve targeted localization with remarkable efficiency (103, 104). Sources of cell membranes include red blood cells, platelets, immune cells, cancer cells, and bacterial membranes. Studies indicate that endothelial cell uptake of extracellular vesicles fused with cell membranes is increased by 2–3 times, and by 5 to 8 times in cardiomyocytes, compared to unmodified vesicles (105). Platelet membrane-modified extracellular vesicles not only mimic the binding properties of platelets and monocytes but also facilitate endosomal escape following macrophage endocytosis. They deliver miRNAs into the cytoplasm and induce a shift from the M1 to the M2 macrophage phenotype. This transition decreases the production of inflammatory factors and consequently aids in cardiac repair (106). Monocyte membrane-decorated MSC also significantly increases homing efficiency to the injured heart and improves treatment outcomes (107).

### 6.2 Engineered exosomes as therapeutic carriers for drug delivery

Traditional drug carriers include synthetic lipid nanoparticles and virus vectors, but their targeting ability and loading capability are relatively limited. As a source of cell nanovesicles, exosome has several merits, including good biocompatibility, stability, targeting, low immunogenicity, which make it a rare natural carrier in the field of drug delivery (16, 17). The comparison of engineered exosomes as therapeutic carriers for drug delivery is provided in Table 2.

**Table 2:** Comparison of characteristics among exosomes, liposomes and viral vectors.

Characteristics	Engineered exosomes	Liposome	Viral vectors
Size	30–200 nm	20 nm up to several micrometers	20–100 nm
Targeting	Good	/	Good
advantages	Preserve the inherent properties of natural exosomes, including good biocompatibility, low toxicity and immunogenicity, stability, and biological barrier penetration ability (16, 17), while acquiring additional or enhanced functions through bioengineering (49)	Flexible modification, sufficient loading and high delivery efficiency (108, 109)	Avoiding immune system detection, self-replication, efficient transduction (110, 111)
Limitations	Multiple challenges remain in their clinical application (112)	Rapid clearance, limited targeting ability (109, 113)	Potential pathogenic, High immunogenicity (111)

**Figure 3:**
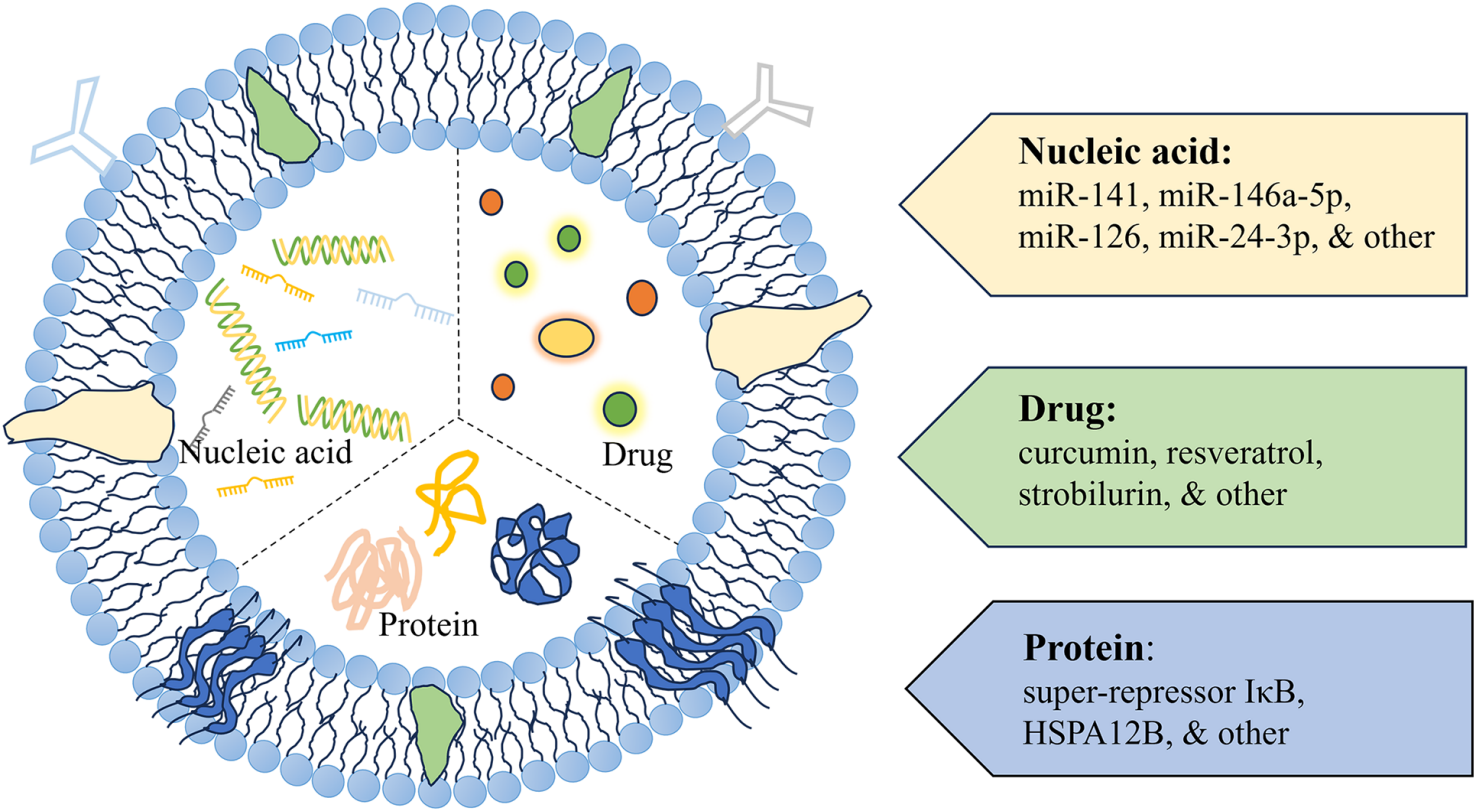
A schematic diagram of exosomes as delivery vehicles. Exosome delivery systems designed for cardiac therapy typically incorporate modifications with nucleic acids, drugs, and proteins.

Engineered exosomes can traverse biological barriers and transport bioactive components (Figure 3). These exosomes are capable of ferrying poorly characterized molecules and drugs, while also evading the P-glycoprotein drug efflux mechanism, which can reduce issues with drug resistance (114, 115). Thus, they are considered natural drug delivery vehicles (116–119).

#### 6.2.1 Using exosomes to load nucleic acid substances such as miRNAs

Research increasingly indicates that microRNAs (miRNAs) are key in driving the healing properties of exosomes (120). The dysregulation of miRNAs is linked to a variety of illnesses, including but not limited to cancer, diabetes, obesity, viral infections, and diseases of the cardiovascular system (121–123). Real and co-authors observed a stark contrast in the miRNA content of exosomes from those with septic shock compared to healthy volunteers. For example, miR-27a levels in exosomes from sepsis survivors were sixfold higher than in controls, suggesting a possible role for exosomal miRNAs in sepsis pathogenesis, including the inflammatory response, oxidative stress, and cell cycle regulation (124). MiRNA-21 has been shown to protect against sepsis-induced cardiac dysfunction, and its upregulation could be a potential strategy for treating septic cardiomyopathy (125). Exosomes can shield miR-21 from RNase degradation and deliver it effectively to target cells, thus decreasing PDCD4 protein levels, reducing apoptosis, and promoting cardiac recovery (80).

Wang et al. demonstrated that miR-223-KO mesenchymal stem cell exosome injection did not improve cardiac function or survival rates in septic mice, highlighting the importance of miR-223 in mediating the protective effects of mesenchymal stem cells on septic cardiomyopathy by downregulating Sema3A and Stat proteins (72). Pei et al. discovered that pre-injecting miR-141-enriched mesenchymal stem cell exosomes could restore myocardial function in a CLP sepsis mouse model, as evidenced by improvements in left ventricular ejection fraction and short-axis shortening (73). Additionally, Liu C et al. confirmed that miR-146a-5p, carried by mesenchymal cell exosomes, has protective effects in cardiomyocytes and myocardial tissue in sepsis models by negatively regulating MYBL1 (74). Zhang et al. also discovered that overexpression of miR-146 in exosomes from bone marrow stromal cells reduced the expression of adhesion molecules during sepsis. This led to a decrease in macrophage and neutrophil accumulation in the myocardium and ameliorated septic cardiomyopathy (75).

Moreover, overexpression of miR-181b has been found to downregulate HMGB1 expression in septic rats, leading to reduced inflammatory factors and myocardial damage, and inhibited cardiomyocyte apoptosis (126). In another study, the cardioprotective effects were attributed to the transfer of miR-181b via exosomes from cardiosphere-derived cells (CDCs) to macrophages, which subsequently reduced PKCδ transcript levels. Notably, while exosomes from fibroblasts alone did not confer protection in this model, those loaded with miR-181b were capable of altering the macrophage phenotype and providing cardioprotection. Conversely, inhibiting miR-181b in CDC exosomes diminishes their cardioprotective properties (81, 127).

In addition, mRNA and other nucleic acid drugs have great potential in the treatment of diseases. However, some characteristics of RNA molecules, such as instability in the body and difficulty in crossing cell membranes and blood-brain barriers, hinder the further application of nucleic acid drugs. Therefore, there are now studies on using exosomes to load mRNA to treat diseases. In innovative research, the LiuM team developed inhalable IL-12mRNA-loaded exosomes IL-12-Exo (128); Kojima et al. focused on the treatment of Parkinson's disease and attempted to deliver catalase mRNA through designed exosomes. to the brain (129); while Wang et al. used exosomes to deliver HChrR6-encoding mRNA to HER2+ cells (130); Similarly, Usman et al. experimented with red blood cell extracellular vesicles filled with Cas9 mRNA and gRNA, targeting the mir-125b-2 site to combat acute myeloid leukemia in MOLM13 cells (131). These pioneering studies highlight the versatility and potential of exosomes as carriers for mRNA delivery in various therapeutic contexts.

Overall, these studies collectively illustrate the transformative potential of exosome-based nucleic acid delivery systems in various medical fields. Continued research and development in this area could lead to significant breakthroughs in the treatment of septic cardiomyopathy, offering new hope for patients. The challenge now lies in optimizing these delivery systems for clinical use, ensuring their safety, efficacy, and scalability for widespread application.

#### 6.2.2 Using exosomes to load proteins

Engineered exosomes can be utilized to deliver proteins, such as HSP60, which function as cytoprotective molecules to alleviate cell damage caused by oxidative stress and inflammatory agents. The study revealed that endothelial heat shock protein A12B (HSPA12B) ameliorates cardiac dysfunction in sepsis and decreases mortality (75, 76). Tu F et al. discovered that exosomes enriched with endothelial cell-derived HSPA12B were shown to inhibit NF-κB activation in LPS-stimulated macrophages. Choi H et al. engineered exosomes (EXPLOR) carrying srIκB, a stable form of IκB*α*, to create immunosuppressive exosomes. These exosomes blocked NF-κB-mediated gene transcription in the nucleus. In a sepsis mouse model, the application of Exo-srIκB effectively decreased levels of inflammatory markers such as TNF-α, IL-1β, and IL-6, be-sides reducing organ damage (77). Additionally, delivering HSP60 and antioxidant enzymes via exosomes has shown promise in preventing cardiomyocyte damage (86).

#### 6.2.3 Using exosomes to load chemical drugs

In treating septic cardiomyopathy, exosomes can be engineered to transport anti-inflammatory drugs or antioxidants directly to the damaged myocardium, thereby reducing the effects of inflammatory mediators and protecting cardiac cells from further injury (16). Kang JY and his colleagues genetically modified the parent cells of extracellular vesicles (EVs) to display a cardiac targeting peptide (CTP) on the exosome surface. They loaded curcumin into CTP-modified EVs, which delivered curcumin specifically to the heart. These curcumin-loaded EVs exhibited increased bioavailability and enhanced cardio-protection (94). They also co-delivered curcumin and miR-144-3p using CTP-EVs, which not only preserved cardiac targeting capabilities but also significantly improved therapeutic outcomes both *in vitro* and *in vivo*. In another study, Zheng et al. used folic acid-functionalized macrophage-derived exosomes to co-load two anti-inflammatory drugs (resveratrol, strobilurin) to inhibit LPS-induced sepsis and protect against Lung function, exosomes showed strong anti-inflammatory and immunosuppressive activities, and multiple administrations significantly enhanced the protective effect and resisted the second hit of LPS (132).

Several drugs have been identified to ameliorate septic cardiomyopathy at the molecular level. Puerarin mitigates inflammation and oxidative stress in myocardial tissues, and inhibits apoptosis and ferroptosis in cardiomyocytes (133). Emodin reduces the inflammatory response and pyroptosis in cardiomyocytes by suppressing the activation of the NLRP3 inflammasome (134). Capsaicin enhances 14-3-3γ-mediated autophagy, alleviating LPS-induced myocardial injury and dysfunction (135). Therefore, we have high hopes for the treatment of septic cardiomyopathy using drugs that are loaded with these beneficial effects on cardiac function.

### 6.3 Using plant-derived exosome-like nanoparticles as therapeutic vehicles

The source of the exosomes greatly influences their effectiveness. Exosomes from inflammatory cells have different biological functions compared to those from mesenchymal stem cells (MSCs). Research has revealed that MSC-derived exosomes have immunomodulatory and regenerative properties akin to MSCs themselves. They can improve the viability of cardiomyocytes post-ischemia/reperfusion injury and exhibit low immunogenicity (78). Numerous studies are currently developing therapeutic drugs based on MSC-EVs (124, 136).

Due to the limited production of exosomes from mammalian cells, researchers have begun to isolate exosomes from fruits or vegetables, such as grapefruits, broccoli, and ginger. These plant-derived exosomes are being explored for treatment of diagnosed diseases (137–139). While the application of plant exosomes for septic cardiomyopathy treatment remains unexplored, current research highlights their ability to deliver a wide range of therapeutic agents—including chemotherapy drugs, siRNA, DNA expression vectors, and proteins—to target cells, demonstrating therapeutic benefits in mouse models (140). Xu XH and his colleagues discovered that exosome nanovesicles derived from ginseng (G-Exos) can serve as efficient and safe carriers for delivering active miRNAs to BMSCs and inducing their differentiation into neural cells. This study has shown promising results in promoting nerve regeneration and repairing conduction function both *in vitro* and *in vivo* (141). Additionally, according to Teng et al., ginger-derived exocrine-like particles contain small RNA that can affect the intestinal microflora of mice, thereby improving intestinal barrier function and reducing the incidence of colitis (138). Ju et al. pointed out that exocrine-like nanoparticles extracted from grape dregs can avoid the degradation of digestive enzymes in mice and promote the proliferation of intestinal epithelial cells after entering the intestine, thus accelerating the recovery of colitis (142).

In conclusion, plant-derived exosome-like nanoparticles may hold therapeutic potential for septic cardiomyopathy. Therefore, further exploration and validation of the role of plant exosomes in septic cardiomyopathy treatment are warranted.

## 7 Discussion

Exosomes, either inherently or as drug delivery carriers, have garnered wide-spread attention in the diagnosis and treatment of sepsis and cardiovascular diseases (25, 143–145), spearheading a new trend in the biopharmaceutical industry. Engineered exosomes, modified through biotechnological techniques, not only retain their original biological properties but are also endowed with specific functions, enhancing their original capabilities. This positions them as potentially valuable in treating septic cardiomyopathy. This review systematically summarizes the research progress of engineered exosomes in septic cardiomyopathy, innovatively discussing their applications in exosome surface modification techniques, as multi-functional drug delivery platforms, and plant exosome-like nanoparticle carriers. It emphasizes the potential of exosomes in delivering small molecules, proteins, and drugs, and summarizes a collection of RNA, proteins, and drugs beneficial for treating septic cardiomyopathy.

However, despite advances in developing engineered exosomes, multiple challenges remain in their clinical application (112). Enhancing the efficiency and purity of exosome culture, isolation, and purification processes is critical. Large-scale production, quality control, improved targeting, and increased drug loading efficiency must be addressed to meet clinical standards (146). Additionally, standardizing production and storage conditions is essential to maintain exosome stability and biological function. Determining suitable delivery methods and ensuring the biocompatibility and safety of these biomaterials in the human body is also required (147).

Further investigative work is crucial to uncover the interaction mechanisms between exosomes and host cells, their distribution, metabolism, and long-term safety (148). Current knowledge gaps regarding exosome dynamics and targeting in different organisms add to the uncertainty of their clinical translation. Researchers are actively seeking solutions to develop engineered exosomes into effective therapeutic tools.

Engineered exosomes are an innovative biotherapeutic carrier with significant potential in septic cardiomyopathy treatment research. They offer modulation of inflammatory responses, enhancement of myocardial repair, and targeted drug delivery. These capabilities suggest engineered exosomes as a promising direction for new therapeutic strategy development.
