# Supplementary material for: Engineered exosomes: a potential therapeutic strategy for septic cardiomyopathy
Source: Front Cardiovasc Med. 2024 Jun 28;11:1399738. doi: 10.3389/fcvm.2024.1399738 (PMC11239395; doi:10.3389/fcvm.2024.1399738)
Supplement: Supplementary file 1 [file Datasheet1.pdf]

Supplementary materials

Table S1: The diagnostic value of exosome contents for sepsis and related diseases in clinical patient studies

Diseases	First sampling time	Sampling time point	Source	Summary of findings	Reference
Septic cardiomyopathy	ICU admission (day 1)	Day 1 and day 3	Neutrophils in peripheral blood	The expression of miR-150-5p is down-regulated	(Ye, Lin et al. 2023)
Sepsis and septic shock	Within 48 hours of study enrolment	Once	Plasma	The total plasma exosome level in sepsis patients is associated with organ failure and mortality.	(Im, Yoo et al. 2020)
Sepsis	Within 24h after ICU admission	Once	Plasma	The combined use of miR-483-3p and let-7d-3p as biomarkers for early diagnosis of sepsis.	(Qiu, Fan et al. 2022)
Sepsis and septic shock	Day of hospitalization	Day of hospitalization (day 0) and day 4.	Serum	The levels of miR-21-5p and miR-193a-5p were positively correlated with the severity of the disease, the survival rate of patients with high level of miR-30a-5p and miR-125b-5p was lower, and the level of exocrine miR-199b-5p was higher than that of healthy volunteers.	(Reithmair, Buschmann et al. 2017)
Sepsis	Within 24h of the diagnosis of sepsis	Once	Serum	The expression of hsa_circRNA_104484 and hsa_circRNA_104670 is down-regulated and has moderate diagnostic value.	(Tian, Liu et al. 2021)
Septic shock	Day 1 from the time of diagnosis	Day 1, day 3 and day 5	Plasma	EV-DNMT mRNAs load, when coupled with total plasma EV number, may be a novel method to diagnose septic shock upon ICU admittance	(Dakhlallah, Wisler et al. 2019)
Sepsis-AKI	Day of hospitalization	Daily during the first week of the admission.	Urine	Urinary neutrophil gelatinase-associated lipocalin (uNGAL) and Urinary exosomal activating transcriptional factor 3 is up-regulated. They are early sepsis-AKI biomarkers.	(Panich, Chancharoenthana et al. 2017)

Table S2 The comparison of the administration methods of engineered exosomes.

Mode of administration	Injection model	Exosome modification mode	Target site	Targeting efficiency	References
Systemic administration	Tail vein injection in rats	Conjugating with CHP through a DOPE-NHS linker.	Ischemic myocardial tissue	Ex vivo labelling of infarcted rat heart sections showed increased retention of CHP-tagged exosomes compared to Scr-tagged exosomes.	(Vandergriff, Huang et al. 2018)
	Tail vein injection in mice	Expressing cardiac-targeting peptide (CTP)-Lamp2b on the exosomal membrane	Cardiac cells	Delivery of CTP-Exo was 16% greater than that of CTL-Exo in H9C2 cells.	(Kim, Yun et al. 2018)
	Tail vein injection in mice	Coating with platelet-membrane	Infarcted myocardial tissue	Endothelial cell uptake of extracellular vesicles fused with cell membranes is increased by 2 to 3 times, and by 5 to 8 times in cardiomyocytes, compared to unmodified vesicles.	(Hu, Wang et al. 2021)
Local administration	Intracoronary infusion or intramyocardial injection in pigs	/	/	Bioluminescence result shows that signal intensity was much higher after in intramyocardial injection when compared with intracoronary infusion of exosomes.	(Gallet, Dawkins et al. 2017)
	Intrapericardial injection in (SD) rats	/	/	MSC-exosomes accumulate in the cardiac-draining mediastinal lymph node (MLN). Exosomes can also be absorbed by epicardial and pericardial cells.	(Zhu, Liu et al. 2022)

References:

Dakhlallah, D. A., J. Wisler, M. Gencheva, C. M. Brown, E. R. Leatherman, K. Singh, K. Brundage, T. Karsies, A. Dakhlallah, K. W. Witwer, C. K. Sen, T. D. Eubank and C. B. Marsh (2019). "Circulating extracellular vesicle content reveals de novo DNA methyltransferase expression as a molecular method to predict septic shock." Journal of Extracellular Vesicles **8**(1): 1669881.

Gallet, R., J. Dawkins, J. Valle, E. Simsolo, G. de Couto, R. Middleton, E. Tseliou, D. Luthringer, M. Kreke, R. R. Smith, L. Marbán, B. Ghaleh and E. Marbán (2017). "Exosomes secreted by cardiosphere-derived cells reduce scarring, attenuate adverse remodelling, and improve function in acute and chronic porcine myocardial infarction."

European Heart Journal **38**(3): 201-211.
Hu, S., X. Wang, Z. Li, D. Zhu, J. Cores, Z. Wang, J. Li, X. Mei, X. Cheng, T. Su and K. Cheng (2021). "Platelet membrane and stem cell exosome hybrid enhances cellular uptake and targeting to heart injury." Nano Today **39**: 101210.
Im, Y., H. Yoo, J. Y. Lee, J. Park, G. Y. Suh and K. Jeon (2020). "Association of plasma exosomes with severity of organ failure and mortality in patients with sepsis." Journal of Cellular and Molecular Medicine **24**(16): 9439-9445.
Kim, H., N. Yun, D. Mun, J.-Y. Kang, S.-H. Lee, H. Park, H. Park and B. Joung (2018). "Cardiac-specific delivery by cardiac tissue-targeting peptide-expressing exosomes." Biochemical and Biophysical Research Communications **499**(4): 803-808.
Panich, T., W. Chancharoenthana, P. Somparn, J. Issara-Amphorn, N. Hirankarn and A. Leelahavanichkul (2017). "Urinary exosomal activating transcriptional factor 3 as the early diagnostic biomarker for sepsis-induced acute kidney injury." BMC Nephrology **18**(1): 10.
Qiu, G., J. Fan, G. Zheng, J. He, F. Lin, M. Ge, L. Huang, J. Wang, J. Xia, R. Huang, Q. Shu and J. Xu (2022). "Diagnostic Potential of Plasma Extracellular Vesicle miR-483-3p and Let-7d-3p for Sepsis." Frontiers In Molecular Biosciences **9**: 814240.
Reithmair, M., D. Buschmann, M. Märte, B. Kirchner, D. Hagl, I. Kaufmann, M. Pfob, A. Chouker, O. K. Steinlein, M. W. Pfaffl and G. Schelling (2017). "Cellular and extracellular miRNAs are blood-compartment-specific diagnostic targets in sepsis." Journal of Cellular and Molecular Medicine **21**(10): 2403-2411.
Tian, C., J. Liu, X. Di, S. Cong, M. Zhao and K. Wang (2021). "Exosomal hsa_circRNA_104484 and hsa_circRNA_104670 may serve as potential novel biomarkers and therapeutic targets for sepsis." Scientific Reports **11**(1): 14141.
Vandergriff, A., K. Huang, D. Shen, S. Hu, M. T. Hensley, T. G. Caranasos, L. Qian and K. Cheng (2018). "Targeting regenerative exosomes to myocardial infarction using cardiac homing peptide." Theranostics **8**(7): 1869-1878.
Ye, R., Q. Lin, W. Xiao, L. Mao, P. Zhang, L. Zhou, X. Wu, N. Jiang, X. Zhang, Y. Zhang, D. Ma, J. Huang, X. Wang and L. Deng (2023). "miR-150-5p in neutrophil-derived extracellular vesicles associated with sepsis-induced cardiomyopathy in septic patients." Cell Death Discovery **9**(1): 19.
Zhu, D., S. Liu, K. Huang, Z. Wang, S. Hu, J. Li, Z. Li and K. Cheng (2022). "Intrapericardial Exosome Therapy Dampens Cardiac Injury via Activating Foxo3." Circulation Research **131**(10): e135-e150.
